# PATients’ views of Health and WORK advice (PATcHWORK) study

**DOI:** 10.1093/occmed/kqag102

**Published:** 2026-09-21

**Authors:** K Lin, R Singhal, V Parsons, K Walker-Bone, G Wynne-Jones, E Godfrey, E Wainwright, I Madan

**Affiliations:** Occupational Health Service, Guy’s and St Thomas’ NHS Foundation Trust, London, SE1 7NJ, UK; London Centre for Work and Health, London, UK; Occupational Health Service, Guy’s and St Thomas’ NHS Foundation Trust, London, SE1 7NJ, UK; London Centre for Work and Health, London, UK; Arthritis UK/Medical Research Council Centre for Musculoskeletal Health and Work, Occupational Health Service Guy’s and St Thomas’ NHS Foundation Trust, London, SE1 7NJ, UK; King’s College London, London, SE1 9RT, UK; School of Public Health and Preventive Medicine, Monash University, Melbourne, VIC 3004, Australia; School of Medicine, Keele University, Staffordshire, ST5 5BG, UK; King’s College London, London, SE1 9RT, UK; Aberdeen Centre for Arthritis and Musculoskeletal Health (Epidemiology Group), School of Medicine, Medical Sciences and Nutrition, University of Aberdeen, Aberdeen, AB25 2ZD, UK; Arthritis UK/Medical Research Council Centre for Musculoskeletal Health and Work, School of Medicine, Medical Sciences and Nutrition, University of Aberdeen, Aberdeen, AB25 2ZD, UK; Centre for Pain Research, University of Bath, Bath, BA2 7AY, UK; London Centre for Work and Health, London, UK; Arthritis UK/Medical Research Council Centre for Musculoskeletal Health and Work, Occupational Health Service Guy’s and St Thomas’ NHS Foundation Trust, London, SE1 7NJ, UK; King’s College London, London, SE1 9RT, UK

## Abstract

**Background:**

A variety of health and work support (H&WS) services are available in the UK, yet little is known about patients’ preferences for such support.

**Aims:**

To develop an understanding of patients’ preferences for H&WS, including the type of support desired, optimal setting, timing and mode of delivery, appropriate providers, integration with existing services and views on employers’ receptiveness to H&WS information.

**Methods:**

We conducted a qualitative study using semi-structured interviews. Participants were recruited through existing research networks and professional societies following informed consent. Interview questions were developed in collaboration with the University of Aberdeen Epidemiology Patient and Public Involvement and Engagement group. Interview responses were analysed using inductive and deductive reflexive thematic analysis.

**Results:**

Fifteen interviews were conducted with participants (11 females, 4 males; mean age 48.9 years), generating six overarching themes. Three themes related to ‘health and work issues’: disability discrimination, lack of H&WS awareness and trust concerns. Three themes identified key ‘H&WS factors’ for employment: flexible working, accessibility to support, and the importance of feeling valued and supported. Participants desired simplified yet holistic and flexible H&WS delivery. Key policy and practice considerations based on insights from this study include improving H&WS publicity through NHS websites and medical waiting areas, integration of work discussions into medical consultations and employer provision of evidence-based health promotion programmes and manager training.

**Conclusions:**

This study provides valuable insights into patients’ preferences about H&WS delivery, highlighting crucial factors for employers and national policymakers to consider, aligned with the recently published Mayfield report.

## Teaser

Preparatory to a quality improvement project, this study interviewed UK patients to hear their views about health and work support (H&WS). There was low awareness about H&WS and disability discrimination. To support people with long-term health conditions to work, they need a simple but accessible and flexible holistic H&WS with more visibility. Patient preferences from this study offer important insights for employers, the NHS, and policymakers to develop improved H&WS delivery.

Key learning points **What is already known about this subject?** 

- Work can support psychosocial and physical health and can be a part of successful rehabilitation from ill-health, so it is vital to support people with LTHCs to resume or remain at work, in line with the NHS *Fit for the future: 10 Year Health Plan.*
- Different models of H&WS delivery have been trialled in the UK, including the ‘train and place’ models of rehabilitation, and the contrasting ‘place and train’ model (e.g. IPS) which is adopted by the current government Connect to Work programme.
- There is limited understanding of patient preferences for H&WS delivery, such as the support type they require, who to receive this from, the timing and modality of H&WS advice, and which services and locations might be best placed to deliver this.

**What does this study add?** 

- The study findings align with *Keep Britain Working* by illustrating the likely importance of partnership between employees, employers and government to help keep individuals with LTHCs at work.
- It offers insight on health and work concerns to address (disability discrimination, H&WS unawareness and trust concerns with managers and H&WS advisors) and identified patient-informed factors to consider when designing H&WS (flexible working, accessibility of support and the importance of feeling valued and supported).
- Participants preferred a simple, holistic H&WS in ‘one place’ with medically trained advisors, and written information on the NHS website; in keeping with the Mayfield report, there is a desire for H&WS at various stages of health and work journey, including evidence-based health promotion and pre-employment support.

**What impact this may have on practice or policy?** 

- Employers are encouraged to review and enhance their H&WS policies, ensuring they are accessible, well-communicated and supported by regular manager training; evidence-based health promotion programmes should be considered, ideally informed by OH to ensure it is appropriate for the workforce.
- To address the lack of H&WS awareness, a simple and unified support in ‘one place’, combined with written information in medical settings and the NHS website could increase visibility and accessibility; clinician training to support incorporation of work discussions in non-OH medical consultations can further raise awareness.
- While the Mayfield report proposed a non-clinical ‘Workplace Health Provision’, this study adds further supports for a national OH service reflecting participants’ preference for a simplified, holistic ‘one place’ H&WS with medically trained advisers; additional clinician training would likely be required to expand the current OH capacity to support this.

## Introduction

Supporting people with health conditions to remain working is crucial since work generally benefits health and wellbeing [1,2], aiding ill-health rehabilitation [3,4].Various UK health and work support (H&WS) strategies for long-term health conditions (LTHCs) have been trialled, including ‘train and place’ [5], ‘place and train’ (e.g. Individualised Placement and Support (IPS) [6], Connect to Work [7]), WORKWELL [8], Access to Work [9] and occupational health (OH). However, patient preferences about H&WS are under-researched, despite being essential for designing effective support and aligning with NHS plans for integrated work, health and skills services [10]. Mayfield’s *Keep Britain Working* review highlighted the need for better support for people with LTHCs to remain at work amid limited evidence of effective practice [11]. This study aimed to explore patients’ preferences regarding H&WS type, setting, timing, delivery mode, provider, integration with existing services and perception of employer receptiveness.

## Methods

This qualitative research involved semi-structured interviews, November 2024 to February 2025, recruiting through research and professional societies.

Participants were UK adults (≥18 years) who, through ill-health, were unemployed, off sick or at risk of job loss. The target pragmatic sample was 24 to maximise diversity [12,13].

Two researchers (KL, RS), both OH physicians unknown to participants, conducted remote interviews using a topic guide informed by study aims and the University of Aberdeen Epidemiology Patient and Public Involvement and Engagement group.

Iterative inductive (from the ground up) and deductive reflexive thematic analysis (applying relevant existing knowledge) followed Braun and Clarke [14]. Researchers (KL, RS) independently double coded verbatim transcripts of interviews before jointly reviewing and refining them to derive themes and subthemes.

Guy’s and St Thomas’ NHS Foundation Trust sponsored this research. King’s College London granted ethical approval. The study complied with the Data Protection Act 2018 and NHS England Code of Practice (Confidentiality).

## Results

Twenty-eight individuals expressed interest, of whom 15 participated (one was ineligible, two withdrew from ill-health and ten were non-contactable). Most were female (73%), White (87%), with a mean age of 48.9 years (Table 1).

**Table 1:** Participant demographic characteristics

Characteristics	*n* (%)
Total number of participants	15
Age (years), mean (SD)	48.9 (10.6)
30–39	4 (27)
40–49	3 (20)
50–59	5 (33)
60–65	3 (20)
Gender	
Male	4 (27)
Female	11 (73)
Ethnicity	
White	13 (87)
Another ethnic group	1 (7)
Prefer not to say	1 (7)
Highest level of education achieved	
College/vocational qualification	3 (20)
Undergraduate degree	5 (33)
Postgraduate degree	4 (27)
Professional qualification	3 (20)
Geographical area	
Northern Ireland	1 (7)
Scotland	3 (20)
England	11 (73)
Employment	
Regular employment full-time	3 (20)
Regular employment part-time	3 (20)
Self-employed	8 (53)
Unemployed	1 (7)
Employment sector	
Health	5 (33)
Hospitality	2 (13)
Third	2 (13)
Architectural	1 (7)
Business	1 (7)
Financial	1 (7)
Welfare	1 (7)
Protective	1 (7)
N/A	1 (7)

Six overarching themes were identified, relating to health and work issues and preferred H&WS features, summarised in Table 2.

**Table 2:** Overarching themes and subthemes

Theme	Subtheme
A. Disability discrimination	Work disability stigma Disability visibility
B. Lack of H&WS awareness	Employee unawareness Employer unawareness Lack of occupational focus in medical consultation
C. Trust concerns	Mistrust between the manager and employee H&WS partiality concern Confidentiality concern
D. Flexible working	Autonomy to modify own work Self-employment
E. Accessibility to support	Simplicity Flexibility Timely support
F. Importance of feeling supported and valued	Implementing adjustments Empathy Employer hypocrisy

Reported adverse consequences of disability disclosure included job loss (quote A1 in Supplementary Table S1) and reduced business opportunities (A3), indicating disability stigma and discrimination. Disability visibility influenced support, so individuals with invisible impairments reported less support (A4) and adjustment envy (A5).

H&WS awareness gaps were noted among employees, employers and medical professionals (B6, B11). Participants reported unawareness of workplace advocates and poorly located workplace policies (B4, B9). Written comprehensive H&WS information was desired (B2). Limited managerial knowledge delayed support (B9), prompting calls for leadership training. H&WS information in medical settings and work discussions in clinical consultations were suggested (B12–13). However, a clinician amongst participants felt insufficiently trained for this (B14).

Mistrust between managers and employees contributed to health condition concealment (A2). Concerns with H&WS impartiality (C2) and confidentiality were common, but mitigated by medically trained advisers (C4), and some valued OH recommendations as credible evidence (C3).

H&WS factors included flexible working. Autonomy over work timings, location and environment appeared vital for sustaining employment, especially with fluctuating symptoms (D1–2, Supplementary Table S2). Self-employment offered autonomy but increased financial vulnerability when unwell (D3–4).

Accessibility of H&WS was important. Participants preferred simple H&WS in ‘one place’ (E1) and information on the NHS website (E2).They valued multiple access modes (in-person, telephone and video) (E3), varied options (e.g. job centres, GPs, medical specialists and self-referrals) (E4–5), timely support (E6), automated GP fit-note referrals (E7), out-of-hours access (E8, E9) and support throughout their health and work journey (E10–12).

Feeling supported and valued was important. Empathy (F5) and implementing adjustments fostered support and continued employment (F1, F2). Discrepancies between work policies and employee experience (F6) suggested ‘employer hypocrisy’. Some perceived well-being programmes as ‘tick-box exercises’ (F7) and ‘puppet for the senior leadership team’ (F8), implying that they were performative.

## Discussion

This study offers insight into patients’ H&WS preferences. Participants preferred a holistic service in ‘one place’ offering work adjustment and financial advice, support-group signposting and pre-employment support, which is well-evidenced with IPS [6]. Written guidance on available H&WS, condition-specific adjustment recommendations and employer discussion advice were desired, ideally on the NHS website for accessibility and publicity. Manager training was emphasized to improve understanding of LTHCs, ensure timely support and reduce disability discrimination, aligning with Learning and Work Institute recommendations [15].

Convenience, medical knowledge, impartiality and confidentiality were important factors influencing preferences for H&WS setting and provider. Participants wanted work discussions during their medical consultations, with H&WS referrals made by clinicians or through self‑referral and wanted automated referrals triggered by GP fit notes, echoing Nye’s report [16].

H&WS throughout the health and work journey was desired, consistent with Mayfield report’s ‘Health Working Lifecycle’ [11]. A key strength of this study is participant diversity across medical conditions, employment status, work sectors and regions. Both interviewers are OH physicians; while this influenced interpretation, it also enabled meaningful contextualisation of participant responses. Limitations include selection bias from voluntary participation since stronger views may be represented. The smaller and demographically narrow sample (predominantly female, White and self-employed) limits transferability. However, according to Malterud *et al*. [17], the case analysis strategy, strong quality of interview dialogue and dense sample specificity support adequate information power.

This study offers important consideration factors for employers and commissioning bodies when designing H&WS. It contributes to the NHS plan [10] and aligns with *Keep Britain Working* on partnership between employees, employers and government to sustain work with LTHCs [11]. Addressing disability discrimination, H&WS unawareness and trust concerns is vital.

Employers should review their H&WS policy accessibility, manager training and collaborate with OH for evidence-based health programmes [11,18].

While the Mayfield report proposed a non-clinical ‘Workplace Health Provision’ [11], this study adds further supports for a national OH service [16] reflecting participants’ preference for a simplified, holistic ‘one place’ H&WS with medically trained advisers. Combined with information in medical settings and the NHS website, this could enhance H&WS visibility and access. Additional clinician training will be essential to achieve a national OH service, since current capacity is limited [11].

Further research is needed to gather perspectives from other key stakeholders, including employers and multidisciplinary practitioners in healthcare.

## Supplementary Material

kqag102_Supplementary_Data
