# Supplementary material for: PATients’ views of Health and WORK advice (PATcHWORK) study
Source: Occup Med (Lond). 2026 Sep 21;76(7):494–8. doi: 10.1093/occmed/kqag102 (PMC13740510; doi:10.1093/occmed/kqag102)
Supplement: Supplementary file 1 [file kqag102_supplementary_data.zip › Patchwork Table S2.docx]

| **Table S2. Themes, subthemes and illustrative quotes related to H&WS factors** | | | |
| --- | --- | --- | --- |
| **Theme** | **Subtheme** | **Illustrative Quote** | **Quote Number** |
| **D. Flexible working** | **(1) Autonomy to modify own work** | “I have fibromyalgia and I have arthritis of my hip which means that I am in pain most of the time. Which can make work a little bit tricky. Early mornings are not good for me because there's still a bit of lag from my overnight medication, so one of the reasons why I enjoy working the way that I am at the moment is that I have flexibility to move things around and to give myself space and time. Between things I obviously there are some things that are more effective,..But yes, the bigger issues are the kind of pain and some degree of brain fog, so have you know sometimes when it's a bit trickier to concentrate on things…” (F, 57) | D1 |
|  |  | “I am in charge of myself and as such in charge of when I see my clients. So although I do, I mean rarely ever cancel a client, because if I do have to get up early or something for a client and I'm knackered and everything's hurting, I would still do that. But obviously I know that maybe the next day I haven't got to see any clients, so I can just not have to worry about getting up or whatever, so definitely affects it but I can work around it quite a lot, which is obviously really helpful compared to like working in office or working…” (F, 56) | D2 |
|  | **(2) Self-employment** | “…when I can't work I then have to pay for an additional staff member, or an additional staff member has to work longer hours because I'm unable to cover that shift. In addition to things like the back of house admin side of the job also gets then delayed, because I may need to take heavy medication to help with the pain management, which means that a lot of pressure falls on to my business partner when I'm unable to sort of function on a day-to-day basis.” (F, 47) | D3 |
|  |  | “I have also lost business because I had a flare up…I couldn't go to some big meetings and then it affected the work from there because they just moved away from me….One of the people I knew said look, it's unfortunate, but you're now not involved because this was the big mid project meeting where we all came together and agreed who was doing what and so since you weren't there, we didn't know if you would be there. We've actually split your responsibilities up and that's what the other one said to me as well. So it has been a bit unfortunate and it's the kind of thing where you work on your own and it's not you work for a big company and you have lots of support, financial support, everything else.” (M, 58) | D4 |
| **E. Accessibility to support** | **(1) Simplicity** | "So many rules or so much is time consuming or waiting. Or you go here for one thing, you go there for one thing, you need to have more so one stop shops where you can go maybe see where you could get help work and accessing in the workplace and training all in one place. It’s going all over the place just to find out lots of little bits of information... I guess like a central, they call it one-stop shop or one-stop place or a central point of contact…" (F, 60) | E1 |
|  |  | “I think sometimes it's useful to have an app or an update website detailing common illnesses and issues that is easy to access…I can access through NHS website… I would say actually it's helpful to have one central resource…I think just sort of succinct information that gets me to the bottom of this is, what this is about and how I can help myself or get further help….if I speak to occupational health and they say A/B and C, I'd probably go back to a website… let me just check this out. Not that I don't trust the professional. I think I just want another second opinion.” (M, 48) | E2 |
|  | **(2) Flexibility** | “My preference would be in person because I think it's maybe easier to demonstrate what you can and can't do … So it would be easier for me to speak to somebody face-to-face, in person, and I think maybe they could make a judgment on what my issues or understand my issues better by seeing me face-to-face.” (F, 62) | E3 |
|  |  | “I think GPs …they do need to be doing it a lot more on lifestyle medicine and a lot more on work and health… I think it would be good because they have access to everybody. So even if you're not seeking work and health support, if you're not going to a job centre and you're not going to a specialist …everybody's got a GP so that is the place where I think everyone could get that you're hitting everybody.” (F, 33) | E4 |
|  |  | “If there was a person who specialised in supporting people to stay in work, it would be useful if you could be referred to them by the GP, if you could be referred to them by, for me, rheumatology or your consultant team. And also if you went to Citizens' Advice, if Citizens' Advice could refer you, that would be really useful and self-refer.” (F, 62) | E5 |
|  | **(3) Timely support** | “ [OH] come back and they say, OK, we have an appointment 26th of Feb at 3:30 and I say well, I'm sorry, I can't do that, I have other sort of commitments and the next appointment was something like 12th March. So, it's not very responsive…” (M, 35) | E6 |
|  |  | “…if I'm off sick and my doctor signed me off… I've got the medical certificate… they've done their bit, because they've ticked the box, saying that I've got a sickness certificate and there needs to be somebody, somewhere who can keep in touch and say right, how are you? This is what you need.…somewhere that you can access further support if you need it.” (F, 60) | E7 |
|  |  | “I think it's important that it's not just like one day in the week when you can contact [H&WS] for two hours. It's the kind of thing where it'll be nice to have it available at the times when people might need that, and that might not be in normal working hours… not in person round the clock.” (F, 38) | E8 |
|  |  | “I think 24/7 having it available on the website or an app. With the health trust I worked for employed staff around the clock who were working on hospital wards out of ours staff. So I think having that information 24/7.” (M, 48) | E9 |
|  |  | “I can imagine at any stage. You know before even an interview process, if you're applying for a job, it's good to know what support there is to make sure that you would find that job suitable for you. Or being able to as you are preparing to do a job, then you need to be able to access that then and then while you're at a job. I just think at any time it is important to be able to have a sounding board to make sure that work suits.” (F,38) | E10 |
|  |  | “I feel like maybe especially for people who are struggling with their mental health and that's prevented them from working, or they need support. I think in that situation, support before getting a job is more important.” (F, 33) | E11 |
|  |  | “I think that preventative stuff could be valuable in a sense that younger people…and you only know this for hindsight when you're older…You know preventative things for people in our office situation, It would have to be done in a way that it's not boring and that people won't pay attention to it. Because if everybody was just sent out an e-mail, make sure you set up properly at your desk… people will just be like, I'm not going to read that….I do think that as well as support for after you've come injured or you you've become ill, that preventative information would be good for all aspects.” (F, 60) | E12 |
| **F. Importance of feeling supported and valued** | **(1) Implementing adjustments** | “I am very, very fortunate that my employer was really good and together, we've put some reasonable adjustments in place…And it is a two-way beneficial agreement- the more flexible work are with me, the more flexible I am with work.” (F, 43) | F1` |
|  |  | “I had a negative working experience. I've worked for myself for a long time and things got a little bit quiet. So I took on some part time work with a local housing association and they were really inflexible and in the end I had to leave because they wouldn't make any adjustment to my hours of working…. They paid to have an occupational health assessment that they completely ignored as well.” (F, 57) | F2 |
|  |  | “…advice on how to start the conversation with your employer. Who to go to. How to frame it and be positive about it, positive as an individual, me going to the company and this is the positive benefit for you as a company. You know, not me, feeling that I'm just draining stuff from the company. There's positivity for everybody in this.” (M, 58) | F3 |
|  | **(2) Empathy** | “But if you don't have a good relationship with your manager, or perhaps they aren't necessarily as understanding, I could see that being a real barrier to you seeking help” (F,38) | F4 |
|  |  | “I think also having a supportive team of people around you, whether that if you're in an office and it's an HR department, your manager…. There's some better understanding means that if you are unable to go to work that day or you need to start later, it's not because you're being lazy ... So I think it's like all of those things are massively important and the same when you own your own business, having somebody that understands the condition, which is my business partner is really helpful because people realise …although I might be fine 80-90% of the time there will be occasions where it does impact me being able to do my day-to-day role. I think where it's challenging is when you don't have that support around you… So they mistake people with autoimmunity as just sort of being lazy or taking unnecessary sick days as opposed to the fact that it can make you extremely tired.” (F, 47) | F5 |
|  | **(3) Employer hypocrisy** | “…so they had a policy on working with people with a disability that all sounded lovely but it wasn't being implemented in practice.” (F, 57) | F6 |
|  |  | “…but the major negative aspect of [the health promotion programme] is, and I really want to stress that, is that when it's been given lip service when it's kind of, “Oh, let's tick the box and I will give everybody a gym membership. We'll put salad in the cafeteria or we'll say we're doing it.” If it seems purely as a tick box exercise, then it has very little impact and people are doing it in a very superficial level. And I've seen that happen. I've seen that happen with some large companies where I've gone in and done audits and said, “Oh yeah, we do all of this healthy stuff now because we've got now a bowl of fruit that people can help themselves to.” So really?” (M, 50) | F7 |
|  |  | “I've seen a lot of well-being washing in my time. I've been in organisations that will plaster online and on social media that they're doing a lot for well-being and they'll say internally they're doing a lot for well-being. But then employees can feel very much otherwise… You're a puppet for the senior leadership team.” (F,33) | F8 |
| Participants are identified by gender (Female F, Male M) and age in years | | | |
