# Supplementary material for: PATients’ views of Health and WORK advice (PATcHWORK) study
Source: Occup Med (Lond). 2026 Sep 21;76(7):494–8. doi: 10.1093/occmed/kqag102 (PMC13740510; doi:10.1093/occmed/kqag102)
Supplement: Supplementary file 1 [file kqag102_supplementary_data.zip › Patchwork Table S1.docx]

| **Table S1- Themes, subthemes and illustrative quotes related to health and work issues** | | | |
| --- | --- | --- | --- |
| **Theme** | **Subtheme** | **Illustrative Quote** | **Quote Number** |
| **A. Disability discrimination** | **(1) Work disability stigma** | “I do regret mentioning [my health condition] because I was treated worse at work for having that condition, but I don't think I changed my work. In fact, I think I was working harder…I would say more than having the physical condition impacted my work. I think it impacted how I was treated and sort of relationship with my line manager really and how people saw me…They pushed me out of my role and then I ended up pursuing an employment tribunal, which I did win.”(F,33) | A1 |
|  |  | "I hid my illness when I first got the job. I hid my illnesses because I just didn't want to be judged on those, wanting to be judged for me, and then later on they could find out about my illnesses. By then they would know me and that wouldn't be an effect." (F,56) | A2 |
|  |  | “I went to one client…he said, “Well, just to let you know, you'll have an absolute nightmare with parking” … And I just said, “Oh, that won't be a problem for me at all, because I've got a blue badge.” And he kind of paused and stopped and thought and said, “Why is that?” And I said, “Well, I've got multiple sclerosis.” ... He said, “Oh, I didn't know that. We don't employ disabled people. Goodbye.”(M,50) | A3 |
|  | **(2) Disability visibility** | "...when you have something that's autoimmune, because it's not physically or visually obvious, people don't really understand what you're going through with it because there's no kind of visual indications to see that you're not really well, so very rapidly back into the job the levels of stress and busyness returns. I would say probably  within a couple of weeks to a month as opposed to say, if somebody had a broken leg and they could see them hobbling around the office with the broken leg in the plaster on. People tend to be a lot more visually aware when there's visual cues."(F,47) | A4 |
|  |  | "...you still then have to deal with other people's opinions...when you're back in that workplace and suddenly you're getting a special chair, but they haven't got a special chair. So that's actually quite more difficult to be honest and also with me like so it's a hidden disability... when I'm at home.. I would just literally look like, you know, a dead person. But once I was back at work again it no one could see it. So again, people obviously have their opinions and are sometimes not so kind about you having extra stuff. That why? Why haven't they got that or whatever." (F,56) | A5 |
|  |  | "That's what younger people are thinking, you're not doing as much as they are, because you've got a health issue… They think you're on lighter duties, for instance, because you've got osteoarthritis or lung disease, some of them wouldn’t be very happy."(F, 60) | A6 |
| **B. Lack of H&WS awareness** | **(1) Employee unawareness** | "I don't know. I'm so used to relying on myself and my own resilience... I don't know what's on offer, so it's very hard to answer that that question in a meaningful way. " (F, 53) | B1 |
|  |  | "I suppose that's the whole thing. If you said to me, “Oh, there's access to work.” And I hadn't heard of it, I'd have been Googling it to find out what it offers and to see if it does support, you know, I don't know if there was free gym membership for people with disabilities, for example…But yeah, if there is other support out there for disabled workers, it'd be interesting. I think a fact sheet produced by yourselves when you're doing this study, will be a very good tangible output. You know at the top of that list you've got access to work and then all the way down it, you've got whatever." (M, 50) | B2 |
|  |  | "I can see where people would fall through the gaps if they don't have a necessarily a supportive workplace or [OH] available as part of their work. Now where else are they going to find it? Is it on the government website? Or is it through the NHS? And I just, I wonder who's who, within whose scope of practise does that lie? " (F, 38) | B3 |
|  |  | “… you do have to really look through all the policies and you have to sort of delve into that … like occupational health don't have their own website hub where you can kind of find every resource you if you want to find out about something it might be that you have to go to Q pulse, which is our kind of policies and documents database for the whole hospital, and then you have to be very specific in your search terms to find exactly what you're looking for. Like the people policy for things like leave, which might include things like sick leave or sick pay, and details about that. But you have to know that you're looking for people policy.”(F, 38) | B4 |
|  |  | "...from personal experience that GPs hold a lot of power and access to certain services or referrals. So if I needed, for example, with my asthma there have been some chest infections in the past and GPs have expedited a chest X-ray for me to get checked out quickly... I think if I  went through the occupational health route to do that, that would take several months" (M, 48) | B5 |
|  |  | "I haven't accessed any services. Being self-employed and mostly working for independent pharmacies, there's no occupational therapy, there's no personnel HR department to go to...I found out from a friend who is also a pharmacist who also has rheumatoid and she's had it for 10 years longer than me, that there is such a thing as anti-fatigue mat. So I knew from her what to ask for. But it is knowing what's available that might be of help. If it hadn't been for her, I would never have known that these things existed. I know of the phrase reasonable adjustments. But I wouldn't know how to find out what are the reasonable adjustments for my condition that would work in my work environment."(F, 62) | B6 |
|  |  | “I think preventative is also good… I know that most organisations do have a well-being stuff and I'm a well-being champion at my work, so I'm able to help other people… But I do think that as well as support for after you've become injured or you've become ill, that preventative information would be good for all aspects.” (F, 60) | B7 |
|  |  | "...maybe some financial advice might be useful, sort of budgeting, if you are self-employed, advice with tax that might benefit you so that you can afford to reduce your hours." (F, 62) | B8 |
|  | **(2) Employer unawareness** | "I said to [my manager], could you please refer me to occupational health? Because I've been waiting all this time to be referred and nothing's been done. So I did eventually get referred...So it was a year later from the prolapse disc happening. And [OH] said that I'm fit to work, but that I did need support at work. So I needed a chair... my [manager] said, Was it them?" [OH] said that, no. So I contacted the disability section of my work and I said, could you help me get a chair? So I was told by them... instead of asking for a chair, I had to ask for a workplace assessment. So I thought this is ridiculous because this is a year that this has been going on. So I resubmitted it again to be told that there was a minimum of a 26-week waiting period for Access To Work to get back to me. And so again I went back to the Disability section and I said, what do I do in the meantime? Because I've no support at all. And they said when you completed the form, did you follow our policy for completing the form? And I said no because no one told me that I had to do that...somebody should have told me that there's a specific way that you have to do it and this is how you do it... But no, no one's told me that. Only found out afterwards, so they have got Access To Work advocates or something at work...So we were fourteen months after the prolapse disc." (F, 60) | B9 |
|  |  | "Once I was diagnosed, I approached HR to find out about occupational health because I'd heard that it was something the office had access to. I didn't really understand what occupational health was or what it did, but I felt that perhaps I might need some sort of support. I was thinking more on a specific sort of physical process as in like maybe a different type of chair or a different type of table or something. I had very little knowledge as to what occupational health actually did. I struggled, I had to really fight to get an appointment. It took me nearly three months for HR to properly understand that I had a health condition that… wasn't just a sore back. I had to get my manager involved to get HR to issue the letter so I could go and see occupational health... The day I went to see occupational health, they immediately signed me off. They were horrified I hadn't been earlier."(F,47) | B10 |
|  |  | "I'm not sure I would necessarily know where to go to access information for an employee if it was for a different condition other than what I have because I understand what's available to me. But don't know that it would be commonplace all business owners to know where to access information to help support their employees. If [OH is] available, I think it could be helpful. I think that was one of the main reasons we've decided to go down the route of offering private medical care is because I'm not sure that anything like that is available, occupational health wise or how I would access that, get myself to be able to access that. I don't know where I'd start with the information on it." (F, 47) | B11 |
|  | **(3)Lack of occupational focus in medical consultation** | “…if the doctor or the hospital was able to say look you've got this condition, particularly if something's chronic because that's where I've had the issue…this is going to a degree keep reoccurring or going to affect me forever in various degrees. I think that's when to just have advice at that stage. From whoever the healthcare professional is.” (58, M) | B12 |
|  |  | “You get seen annually by the rheumatology, unless you've got an issue. It would be great if in that kind of review, it would be, you know fresh sheet of paper: Are you getting all the support you need? Have you thought about this? Because you only know what you know. And then you can kind of relay that information back to your employer.”(F, 43) | B13 |
|  |  | “Presumably, if you're under a secondary care like a psychiatrist, it would be helpful for them to give some advice. But as the psychiatrist who's being asked about those things, a lot of the time, what I'll say is well, then you have an occupational health specialist because sometimes, especially when I do private work and I know I'm now answering this as a psychiatrist rather than the patient in this interview, a lot of the time, you're like, well, I'm not an occupational health consultant. I can tell you what I think is the person I'm seeing in front of me is a very stressed person, so I would assume you're not ready to go back. But I have no formal training in terms of altered duties or reduced hours or, you know, I sort of make an educated guess.” (M, 35) | B14 |
| **C. Trust concerns** | **(1) Mistrust between the manager and employee** | "… the job that I got without them knowing that I had any health issues. But once I'd been there a year and they wanted to take me on permanently and obviously by then I was probably struggling a lot more as well. Slowly but surely, it came out about my health and … they like sent me to their occupational health. Just I think maybe on their part it was a little bit of like let’s confirm is she lying? I don't know…but it was a tiny part of me thought like, because I'd got the job and then not told them and then I did tell them and then they sent me to occupational health. It was like they wanted a doctor or someone on their side that would actually say, ‘yeah, she has got these things’ or whatever." (F, 56) | C1 |
|  | **(2) H&WS partiality concern** | "The occupational health are often considered to be on the side of the employer. That wasn't my experience of them, but so whether they would be a source that people would feel confident and Just have enough trust in to access it that way." (F, 57) | C2 |
|  |  | “[OH] were neutral, so they didn't side with management, which was what every other senior leader was doing. … I felt like I was ganged up on because every senior manager bar probably one or two, was siding with each other and it became like I had nowhere to go … Occupational health always took the condition for the condition and took the employee for that condition and they didn't treat them any differently so they just wrote about what they saw and then they made it very clear in their notes. So it was a good form of evidence as well to this is the condition I have and this is the support that I should be having…they offered good recommendations and they listened and they were neutral.” (F, 33) | C3 |
|  | **(3) Confidentiality concern** | “I think for medical professionals, that's fine because whilst they might not be specialists in those areas, they have a good grounding on health conditions, etc, whether they're a nurse or a doctor. But I think for a non-medical person to view your health records and perhaps make conclusions and one and one, does it equal two? I'm not sure. I'd have the confidence that they'd be the right person to do that because do they know any more than me? I think those kind of suggestions and kind of thought processes should be medical professionals offering medical advice.” (F, 43) | C4 |
| Participants are identified by gender (Female F, Male M) and age in years | | | |
